# Towards a Formative Assessment of Classroom Competencies (FACCs) for postgraduate medical trainees

**DOI:** 10.1186/1472-6920-8-61

**Published:** 2008-12-17

**Authors:** Christopher S Gray, Anthony J Hildreth, Catherine Fisher, Andrew Brown, Anita Jones, Ruth Turner, Leslie Boobis

**Affiliations:** 1 The Education Centre, City Hospitals Sunderland Foundation Trust, Kayll Road, Sunderland, Tyne and Wear, SR4 7TP, UK; 2 School of Clinical Medical Sciences, Newcastle University, Framlington Place, Newcastle upon Tyne, NE2 4HH, UK; 3 School of Health, Community and Education Studies, Northumbria University, Coach Lane, Benton, Newcastle upon Tyne, NE7 7XA, UK

## Abstract

**Background:**

An assumption of clinical competency is no longer acceptable or feasible in routine clinical practice. We sought to determine the feasibility, practicability and efficacy of undertaking a formal assessment of clinical competency for all postgraduate medical trainees in a large NHS foundation trust.

**Methods:**

FY1 doctors were asked to complete a questionnaire to determine prior experience and self reported confidence in performing the GMC core competencies. From this a consensus panel of key partners considered and developed an 8 station Objective Structured Clinical Examination (OSCE) circuit to assess clinical competencies in all training grade medical staff... The OSCE was then administered to all training grade doctors as part of their NHS trust induction process.

**Results:**

106 (87.6% of all trainees) participated in the assessment during the first 14 days of appointment. Candidates achieved high median raw percentage scores for the majority of stations however analysis of pre defined critical errors and omissions identified important areas for concern. Performance of newly qualified FY1 doctor was significantly better than other grades for the arterial blood gas estimation and nasogastric tube insertion stations.

**Discussion:**

Delivering a formal classroom assessment of clinical competencies to all trainees as part of the induction process was both feasible and useful. The assessment identified areas of concern for future training and also served to reassure as to the proficiency of trainees in undertaking the majority of core competencies.

## Background

The legal standard for a qualified doctor is that of the ordinary reasonable professional exercising skills appropriate to the role and profession [1]. The legal standard takes no account of inexperience nor the learning curve required to achieve clinical competence and confidence.

The development of competence is an acquired skill overseen by an educational and clinical supervisor in the workplace. Whilst junior doctors may develop competence during their training, it is clear that 'out of hours' the designated educational and clinical supervisors are unlikely to be present and many supervisory functions are delegated formally or informally.

It is assumed that newly qualified or newly appointed doctors bring to the work place core clinical skills as defined by the GMC and assessed in their undergraduate curriculum. Whilst the GMC are responsible for defining and overseeing undergraduate curriculum design and delivery, there is inevitable variation between institutions in delivery and assessment. It is likely therefore that there will be variation between graduates within and between institutions.

Clinical training involves the cumulative acquisition of skills and knowledge and competency is achieved and consolidated through supervision and repetition. Against such a background there is the need to ensure that all medical staff are competent in medical procedures and practice commensurate with their career grade. The proposition that such skills can be acquired in a formative way can no longer be defended if the trainee is expected to deliver them in routine practice from day one of employment.

Against this background, the aims of this study were:

1. To determine the feasibility of undertaking a routine assessment of core competencies as part of the routine induction process for postgraduate medical trainees.

2. To provide the host organisation with an overview of GMC defined core competencies in training grade medical appointees.

3. To develop a classroom based assessment of core clinical competencies as a tool for assessing and developing clinical skills.

## Methods

In May 2007 City Hospitals Sunderland NHS Foundation Trust issued an instruction to the Medical Education department that it wished all new medical appointees: foundation year (FY) 1,2, specialist trainee (ST) years 1–3, specialist registrars and Fixed Term Specialist Trainee Appointments (FTSTA) to undertake an assessment of clinical competencies prior to undertaking clinical duties. Responsibility for the assessment was adopted by the trust medical education department. The detail of the assessment was not specified, nor was a measure of or threshold for achieving competence defined.

City Hospitals Sunderland is a 4 star, first wave foundation hospital serving the population of Wearside, North East England. At the time the study was conducted the trust employed 121 trainees (FY 1–2, ST 1–3, FTSTA and specialist registrars. A panel of stakeholders was established to develop the assessment including the trust medical director, clinical tutor, foundation tutor, Newcastle University undergraduate clinical sub-dean, senior nurse teaching staff and foundation programme representatives. We used the GMC recommendations on undergraduate training to inform discussions [2].

### Principles of the assessment

The panel determined that an assessment against an objective statistical standard could not be realistically pre-specified. It was further agreed that as a principle, the assessment should be used to identify areas of strength and weaknesses within a formative framework from which the trainee could develop appropriate clinical competencies whilst supervised in the workplace. Whilst the proposed assessment would be conducted as an objective structured clinical examination (OSCE), there would be no overall pass/fail threshold (either prespecified or to be determined). Recognising the limitations of classroom assessments of competency and in an attempt to move away from the 'E' (examination) element of the OSCE, the assessment was renamed as the Formative Assessment of Classroom Competencies (FACCs).

In keeping with the formative nature of the assessment, results would be shared with the trainee and their educational supervisor from which an agreed training and personal development strategy would be derived. Recognising that competency in the assessment may not necessarily equate with clinical competency, educational supervisors would be encouraged to review the results of the assessment with the trainee in a clinical and career oriented context.

For the host organisation, we specifically sought to determine whether information gained from the assessment could be used to determine whether there are sub groups of trainees for whom significant additional training needs are required and what support would be necessary to enable new appointees to safely develop clinical skills and practice.

### Preparedness for practice; defining the undergraduate experience and confidence in core clinical competencies

Prior to implementing the assessment, a letter describing the aims and objectives of the proposed FACCs was given to all existing FY1 doctors in the trust. These trainees, who were all in the final 4 weeks of their F1 programme, were then asked to complete a questionnaire reporting their own personal experience of the common practical procedures that a newly qualified graduate is expected to perform safely and effectively. Trainees were asked to record the estimated total number of times they had undertaken each task on training mannequins or patients prior to graduation. The FY1 doctors were also asked to self rate their confidence in the core competencies prior to F1 training (retrospective) and currently (final 4 weeks of F1 training) using a Likert scale (1–10 where 1 = no confidence and 10 = confident, no concerns). Finally, FY1 trainees were asked to self rate their preparedness for practice by responding to the question: 'Do you feel your undergraduate training adequately prepared you for undertaking the GMC core practical procedures as listed?' using a Likert scale (0 = totally unprepared, 10 = totally prepared)

### Development of the FACCs

The panel identified common clinical procedures that all medical trainees would be expected to be able to undertake and which could potentially expose both patients and the host organisation to risk. We specifically excluded those learning outcomes and competencies that were more appropriately examined in the finals MBBS examination. Having considered the 22 GMC core clinical and practical skills and analysed the responses from the FY1 questionnaire, a circuit of 8 stations (1 written, 7 observed) was established. Stations were chosen to reflect routine clinical tasks with the emphasis on procedures junior doctors may be called upon to undertake either routinely or out of hours without supervision, or in clinical emergencies whatever their host departmental specialty.

The eight stations chosen comprised: venesection, nasogastric tube insertion, male catheterisation, cannula insertion, prescribing, intermediate life support (including defibrillation), ECG recording, arterial blood gas sampling. Training mannequins were used for all procedures other than ECG recording.

Candidates were given written instructions (including an abbreviated case history) at each station, with a maximum of 7 inclusive minutes to undertake the tasks. Allowing for 30 second transitions between stations, one complete circuit of the FACCS was planned to take a maximum of 1 hour. Prior to roll out of the assessment, a pilot circuit involving 7 outgoing FY1 doctors was conducted to confirm timing, acceptability and practicability.

Candidate performance at each station was assessed by a senior clinician or nurse (who had previously undergone OSCE examiner training) against pre-defined domains assessing infection control techniques; ability to adequately select, prepare and safely dispose of equipment, and the ability to sequence and complete the procedure. Communication skills were not evaluated. For each domain within the station, candidates were scored as having completed the tasks or not. The panel also pre-specified 'critical' domains within each station, i.e. those tasks which if not completed correctly or omitted would expose the patient, staff or the host organisation to clinical risk either directly or indirectly, e.g. hand washing, failure to check equipment or sharp disposal. For resuscitation it was agreed that any error or omission should be defined as critical. On completion of the circuit, candidates were immediately debriefed by the assessor at their final station who advised them of their overall performance and any necessary course of immediate remedial action where appropriate. Results were to be forwarded to each trainee's educational supervisor to inform the initial educational meeting.

The prescribing station comprised a written ward round scenario from which the candidate was asked to prescribe the specified drugs on a standard hospital prescription chart. In order to replicate clinical practice, drugs were specified in a mix of generic and non generic forms, with and without units of dosage. A British National Formulary was available. Domains assessed for the station included: documentation of patient identification, allergies and the prescription of 7 drugs, 2 urgent once-only intravenous preparations, 3 routine and 2 as required. The station was marked by a consensus panel of assessors who determined that any prescription with an error of any of: dosage, units, legibility, was not signed (including block capitals) or dated, was deemed non dispensable. Details of the prescribing assessment will be reported elsewhere.

#### Participants

Completion of a circuit of the FACCS was compulsory for all training grade doctors in the host organisation. The assessment was conducted as part of the routine trust induction process in August 2007. Trainees unable to attend were given an appointment for an alternative circuit 2 weeks later.

#### Analysis

All data was collected on standard proformas and analysed using SPSS Version 14.0. Data are presented as overall means (sd) or medians (IQR) for performance on each station or for each assessment domain where appropriate.

## Results

### Defining the undergraduate experience and confidence in core clinical competencies: the pre FACCS questionnaire

The questionnaire was sent to 25 FY1 doctors, 18 (72%) of whom responded (5 were on annual leave). The retrospective self rated confidence (1–10) in undertaking the core competencies pre FY1 ranged from means of 3.2 (life support) to 8.1 (venesection) (table 1).

**Table 1:** Foundation year 1 doctors' self confidence ratings for core competencies prior to (retrospective) and current (final 4 weeks) of F1 training.

Core competency	Confidence prior to undertaking FY1 (mean, SD)	Confidence at end of FY1 (mean S.D.)	Paired t-test, p-value	95% CI
Venous cannulation	6.7 (3.0)	9.8 (0.5)	<0.0001	1.8–4.5

Arterial blood gases	6.3 (3.3)	9.7 (0.5)	<0.0001	1.9–4.9

Prescribing	4.6 (2.1)	9.5 (0.6)	<0.001	4.0–5.9

Male catheterisation	5.2 (2.9)	8.9 (1.7)	<0.001	2.4–5.0

ECG	7.6 (1.9)	9.0 (2.0)	0.014	0.3–2.5

Venesection	8.1 (2.3)	9.9 (0.3)	0.003	0.7–2.9

Intermediate Life support	3.2 (2.6)	7.1 (2.5)	<0.001	2.8–5.1

Nasogastric tube insertion	5.2 (2.9)	9.3 (1.2)	<0.001	2.6–5.6

Range of scores from Likert scale 1–10 where 1 = no confidence and 10 = confident, no concerns. N = 18.

**Figure 1:**
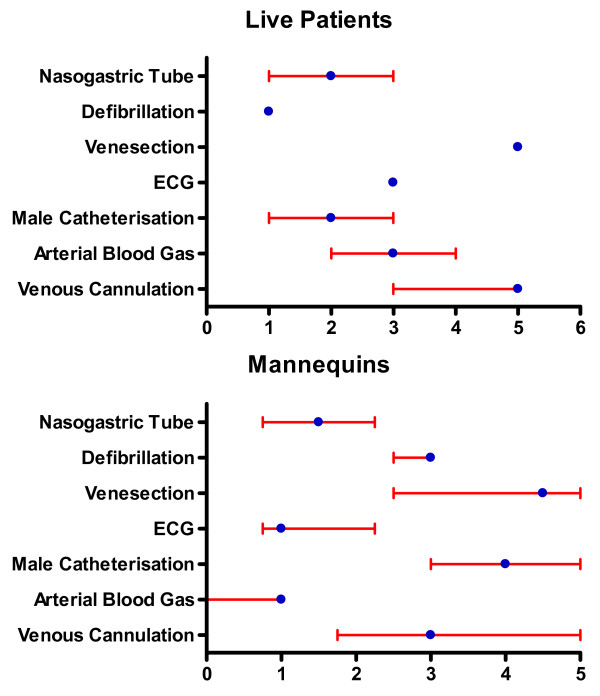
**Median and IQR self reported frequencies of undergraduate experience in the core clinical skills for 18 FY1 doctors**.

By the end of FY1, respondents' confidence in all tasks had increased to a level > 8, with the exception of life support (table 1). There were significant increases in confidence for all competencies. For each of these competencies the median number of times FY1 doctors reported having previously undertaken the procedures on either patients or a training mannequin was 5 or less (figure 1).

**Figure 2:**
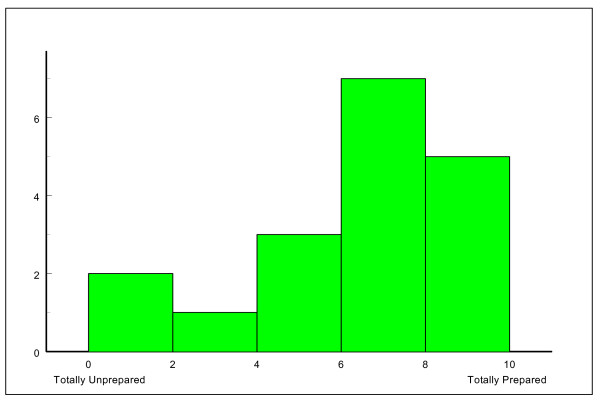
**Self rated reporting of preparedness for practice by FY1 doctors in response to question: 'Do you feel your undergraduate training adequately prepared you for undertaking the GMC core practical procedures as listed?'**.

When asked (via a 10 point visual analogue scale) the question: 'do you feel your undergraduate training adequately prepared you for undertaking the GMC core practical procedures as listed?' the 18 FY1 respondents recorded a median score of 7.0 (IQR 5.0 – 8.0) (Figure 2).

### The Formative Assessment of Clinical Competencies: Practicability

A total of 106 doctors (87.6% of 121 overall) completed the assessment over 14 circuits (6 parallel circuits and 2 stand alone) during the first 14 days of employment in the trust. The majority of these (91 [85.8%]) were achieved in the first three days as part of the routine induction process. There were 29 FY1, 13 FY2, 49 SHO, FTSTA and Specialty Trainee year 1–3 doctors, and 15 specialist registrars. The assessment was hosted in a purpose built corridor of the host organisation's education centre with delivery of each circuit requiring nine assessors/assistants, three volunteer patients and a circuit co-ordinator.

### Candidate performance

**Figure 3:**
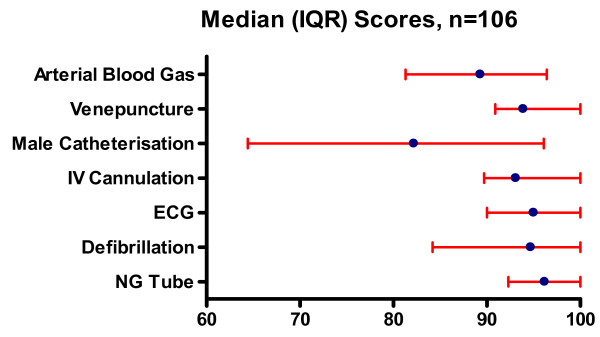
**Distribution of FACCS scores for each station (Median and IQR 1^st ^and 3^rd ^quartiles (all candidates N = 106)**.

For the purpose of this analysis, performance on all stations excluding the prescribing station is reported. For all stations a raw percentage score was derived for each candidate's performance. The overall median scores and their distribution for each station are shown in Figure 3. In order to establish where clinical risk may exist, the frequencies of critical errors/omissions achieved by all candidates within each station were recorded as a percentage of the total number of criticals assessed (table 2).

**Table 2:** Number of 'critical' errors and omissions assessed within each station

Criticals (%) obtained within each station												
Station (max no. of criticals obtainable)	0	1	2	3	4	5	6	7	8	9	10	11

Defibrillation (18)	27.8	32.1	22.8	9.5	1.9	1.9	1.0	0.0	0.0	1.0	1.0	1.0

ECG (11)	77.4	11.3	2.8	2.8	1.9	1.9	1.9	0.0	0.0	0.0	0.0	0.0

Arterial Blood (7)	34.0	38.7	20.8	5.7	0.9	0.0	0.0	0.0	*	*	*	*

Male Catheter (7)	83.0	10.4	3.8	1.9	0.0	0.0	0.9	0.0	*	*	*	*

IVC (6)	58.5	32.1	8.5	0.9	0.0	0.0	0.0	*	*	*	*	*

Venepuncture (5)	95.3	4.7	0.0	0.0	0.0	0.0	*	*	*	*	*	*

NG Tube (5)	84.0	14.2	1.0	1.0	0.0	0.0	*	*	*	*	*	*

(Station criticals) and the performance of all candidates (n = 106) expressed as a percentage of possible criticals for each of the 7 stations.

The range of median scores varied from 82.2 for male catheterisation to 96.2 for nasogastric tube insertion. The 75^th ^percentile was at 100% for all stations apart from male catheterisation (96.1%) and arterial blood gas (96.4%). The lowest value for the 25^th ^percentile was 64.4% for male catheterisation, the other stations ranging from 81.25% to 92.3% (nasogastric tube insertion)

Across all stations there were a small and variable number of errors and omissions for assembling equipment, sequencing and hand washing and aseptic technique, e.g. failure to wear apron/gloves as per local policy. In order to identify specific areas of concern/difficulty we undertook a detailed scrutiny of scores within each station.

The venepuncture, and nasogastric tube stations were completed with the best overall median scores and lowest number of criticals obtained (table 2, figure 3). For the male catheterisation station there was a wide range of overall scores that identified problems with aseptic technique, sequencing and overall familiarity with the process including the failure to comment on repositioning the foreskin in 7.5% of candidates. In contrast however, 83% of candidates scored no criticals.

For the arterial blood gas station, although the overall median score was 89.3%, 66% of candidates scored one or more criticals; 56.6% of candidates failed to check the expiry date on the pre-filled syringe, 17% failed to expel excess heparin/air prior to arterial stab and 21.7% failed to express excess air post procedure prior to capping the syringe. Two candidates failed to dispose of the sharps appropriately.

The high overall median score obtained for the ECG station did not reflect the fact that 23% of candidates scored criticals for this station. These were due to incorrect positioning of the leads and failure to adequately document date, time and any current symptoms on the recording.

For the IV cannulation station, despite an overall median score of 93.1% there was a high (42%) rate of critical errors. This was largely accounted for by the 38.7% of candidates who failed to confirm the content of the saline flush with a second person or comment to that effect before flushing the cannula.

Candidates scored an overall median (IQR) of 94.7% (84.2 to 100.0) on the resuscitation station. In contrast, analysis of the results revealed one or more errors and omissions in 72.2% of candidates (all of which were pre defined as critical). Areas of concern included incorrect clearing of the mouth (17.9%), failure to open the airway (33%), call for assistance (12.3%), remove free flowing oxygen if present (21.7%) and warn to stand clear before defibrillation (6.6%). Candidates who obtained any critical in this station were offered early remedial training for which confirmatory evidence of participation was sought by the clinical tutor.

### Influence of training grade status and distribution of criticals

Analysis was undertaken to compare performance between training grades using Kruskal Wallace ANOVA (Table 3). There were significant differences (adjusted for multiple comparisons) between grades for nasogastric tube insertion where F1s scored significantly higher than F2/SHO/FTSTA grades (p = 0.012), and for arterial blood gas estimation where F1s scored significantly higher than F2/SHO/FTSTA grades (p < 0.001) and STs/registrars (p < 0.001).

**Table 3:** Performance scores (percent) for each clinical station (median, IQR) by training grade.

Station	F1s	F2 SHO and FTSTA, Locums	Registrars
Defibrillation	94.7 (84.2 – 100.0)	89.5 (84.2 – 94.7)	94.7 (88.2 – 100.0)

ECG	95.0 (90.0 – 100.0)	100.0 (90.0 – 100.0)	97.5 (90.0 – 100.0)

Male Catheter	82.2 (64.4 – 100.0)	84.4 (64.4 – 93.3)	83.3 (64.4–97.8)

IVC	96.6 (86.2 – 100.0)	93.1 (89.6 – 100.0)	93.1 (89.6 – 100.0)

Venepuncture	97.0 (93.9 – 97.0)	93.9 (90.9 – 100.0)	97.0 (93.2 – 100.0)

NG Tube	100.0 (96.2 – 100.0)	96.2 (88.5 – 100.0)	96.2 (91.3 – 100.0)

Arterial Blood	96.4 (92.9 – 100.0)	89.3 (78.6 – 92.9)	87.5 (78.6 – 92.9)

## Discussion

In this study we sought to determine whether it was feasible to undertake a formal assessment of clinical competencies in trainee medical staff and whether this could be used to identify and address risk to patients and the host employer.

It has previously been reported that junior medical staff do not feel prepared for the skills they need in the first year of employment [3,4]. Furthermore, studies have highlighted the gap between a trainee's self reported overestimate of level of confidence and their formally assessed performance [5]. It is probable that this confidence/competence gap is influenced by personal factors with evidence from postgraduate trainees suggesting that self reported confidence is linked to characteristics of the trainee including general attitudes and professionalsism [6].

Prior to delivery of the FACCs, we identified some unexpected areas of concern that extend beyond the host organisation. As evidenced in the pre study questionnaire to the outgoing FY1 trainees, not only do junior medical staff report a lack of confidence in performing a range of simple clinical tasks, they have also had little reported experience undertaking and consolidating skills in these tasks on either training mannequins or actual patients prior to practice. Of note is that, apart from venesection, the retrospective self reported confidence in undertaking the practical procedures at the start of F1 training was low. Confidence was lowest for resuscitation and remained low despite completion of foundation training. Such a finding is not new. Evidence suggests that whilst doctors are likely to feel inadequately trained on practical procedures, nursing staff in contrast are more likely to have received formal teaching and supervision when performing clinical techniques [7].

We accept that retrospective recall of both core skill frequency and confidence rating is subject to the limitations of memory recall and potential bias of newly qualified doctors wishing to demonstrate experience, confidence or improvement during training. Furthermore, some doctors may wish to emphasise the limitations of their undergraduate experience. Within the time frame in which this evaluation was conducted it was not possible to prospectively collect such data that would have permitted a more valid interpretation of such findings.

Whilst in principle this was an evaluation of a new assessment, in reality it was a feasibility study, conducted within a short period of time from concept to delivery and during which we had little time to consider what our definition of competency would be. Whilst the overall median scores suggest a general level of competence in excess of 90% for most stations, this does not provide evidence of where problems may arise in clinical practice. The analysis of critical errors provides more useful information that can inform clinical training and supervision. Within the critical domains there is inevitably a hierarchy of risk to the patient, staff and organisation, which we did not attempt to explore in this study. This risk may be direct or indirect and the liability personal or vicarious. Nevertheless we have shown that it is possible to assess trainee medical staff as part of an induction process and identify some areas of risk and training requirements.

The timing of such an assessment is critical; the immediate availability of training grades to on call rotas meant that it was not possible to assess every trainee as part of the induction process during the first 3 days. Despite this we were able to assess 87.6% of trainees with the minimum disruption to service delivery.

The FACCs was conducted as part of the trust induction process and immediately followed lectures on infection control. It is interesting to note therefore that errors still occurred with adherence to infection control techniques suggesting that host organisations may wish to give further consideration as to how mandatory training should be delivered.

Some elements of the FACCs have generalised relevance; performance on the resuscitation station demonstrated that annual training and certification does not necessarily translate into clinical competence and safe practice. This finding is of particular relevance to host organisations that rely on annual CPR training as evidence of competency.

The FACCS was administered to a wide spectrum of trainee medical staff across both training grades and specialities. Interpretation of the overall results for each station must take into account not only the lack of actual clinical experience for FY1 doctors in the first 72 hours of their new career but also the potential for deskilling in some grades and specialities where certain tasks are not routinely/ever undertaken, e.g. Ophthalmology and blood gas/nasogastric tube insertion. Furthermore, it is probable that the unrealistic setting of a classroom assessment and candidate attitudes will have also influenced performance. The performance of the FY1 doctors may in part be accounted for by a focussed undergraduate training and familiarity with the OSCE process. Accepting these limitations however, their performance in comparison with their peers is reassuring and suggests not only the overall competency of the grade but also adequacy of their undergraduate training.

Within the 7 clinical stations however, there are domains where a host organisation and patients would expect absolute competency of all medical staff and for which errors and omissions would have implications for patient safety.

## Conclusion

This study has identified that competence and confidence are inexorably linked and require consolidation of skills through repeat performance. Infrequently performed clinical procedures will inevitably lead to deskilling and it is inappropriate to expect all medical staff to perform all skills competently unless they are regularly practising them. To this extent it is likely that annual training is inadequate to achieve competency and in cases such as defibrillation it seems probable that frequent refresher training across each training grade responsible for resuscitation is necessary to maintain clinical competence.

As an exploratory project the FACCs has limitations and we seek to further develop the circuit construct, the critical domains and scoring system. In practice further consideration needs to be given to how individual performance and results may be rapidly conveyed to both trainee and supervisor and how these may be used in the educational process. Following the study we have had contact from both trainees and educational supervisors seeking detailed feedback on performance in the FACCs. We now seek to further develop the assessment tool and its application in routine practice.

## Competing interests

Professor Gray holds an honorary contract with City Hospitals Sunderland NHS Trust; all other authors are employees of the Trust. There are no other competing interests to declare.

## Authors' contributions

CSG, AJH, CF, AB, AJ, RT, LB contributed to the design and delivery of the study. CSG and AJH undertook the analysis of the study and first draft of the manuscript. AJH provided detailed analysis and production of graphics. CSG, AJH, CF, AB, AJ, RT, LB all contributed to the interpretation of data, writing and review of the final manuscript. All authors read and approved the final manuscript.

## Pre-publication history

The pre-publication history for this paper can be accessed here:



## References

[B1] Bolam v Friern Hospital Management Committee [1957] 2 ALL ER 118.

[B2] General Medical Council (2003). Tomorrow's Doctors. Recommendation on undergraduate training. http://www.gmc-uk.org/education/undergraduate/undergraduate_policy/tomorrows_doctors.asp.

[B3] Hannon FB (2000). A nation al medical education needs' assessment of interns and the development of an intern education and training programme. Medical Education.

[B4] Han WH, Maxwell SRJ (2006). Are medical students adequately trained to prescribe at the point of graduation? Views of first year foundation doctors. Scottish Medical Journal.

[B5] Barnsley L, Lyon PM, Ralston SJ, Hibbert EJ, Cunningham I, Gordon FC, Field MJ (2004). Clinical skills in junior medical officers: a comparison of self reported confidence and observed competence. Medical Education.

[B6] Kaduszkiewicz H, Wiese B, Bussche H van den Self reported competence, attitude and approach of physicians towards patients with dementia in ambulatory care.: Results of a postal survey. http://www.biomedcentral.com/1472-6963/8/54.

[B7] Mason WTM, Strike PW (2003). See one do one, teach one – is this how it still works? A comparison of the medical and nursing profession in the teaching of practical procedures. Medical Teacher.

